# Inhibition of IRAP Enhances the Expression of Pro-Cognitive Markers Drebrin and MAP2 in Rat Primary Neuronal Cells

**DOI:** 10.3390/ijms252212016

**Published:** 2024-11-08

**Authors:** Frida Stam, Sara Bjurling, Erik Nylander, Esther Olaniran Håkansson, Nicholas Barlow, Johan Gising, Mats Larhed, Luke R. Odell, Alfhild Grönbladh, Mathias Hallberg

**Affiliations:** 1 The Beijer Laboratory, Department of Pharmaceutical Biosciences, Neuropharmacology and Addiction Research, Biomedical Centre, Uppsala University, P.O. Box 591, SE-751 24 Uppsala, Sweden; frida.stam@uu.se (F.S.); sara.bjurling@uu.se (S.B.); erik.nylander@uu.se (E.N.); alfhild.gronbladh@uu.se (A.G.); 2 Department of Medicinal Chemistry, Biomedical Centre, Uppsala University, P.O. Box 574, SE-751 23 Uppsala, Sweden; esther.olaniran.hakansson@ilk.uu.se (E.O.H.); luke.odell@ilk.uu.se (L.R.O.); 3 The Beijer Laboratory, Science for Life Laboratory, Department of Medicinal Chemistry, Biomedical Centre, Uppsala University, P.O. Box 574, SE-751 23 Uppsala, Sweden; nicholas.barlow@monash.edu (N.B.); mats.larhed@ilk.uu.se (M.L.); 4 Monash Institute of Pharmaceutical Sciences, Monash University, Parkville, VIC 3052, Australia

**Keywords:** cognition, cortex, dendritic spines, hippocampus, insulin-regulated aminopeptidase (IRAP), microtubule-associated protein 2 (MAP2), primary cell cultures

## Abstract

The insulin-regulated aminopeptidase (IRAP; oxytocinase) is part of the M1 aminopeptidase family and is highly expressed in many tissues, including the neocortex and hippocampus of the brain. IRAP is involved in various physiological functions and has been identified as a receptor for the endogenous hexapeptide Angiotensin IV (Ang IV). The binding of Ang IV inhibits the enzymatic activity of IRAP and has been proven to enhance learning and memory in animal models. The macrocyclic compound 9 (C9) is a potent synthetic IRAP inhibitor developed from the previously reported inhibitor HA08. In this study, we have examined compound C9 and its effects on cognitive markers drebrin, microtubule-associated protein 2 (MAP2), and glial fibrillary acidic protein (GFAP) in primary hippocampal and cortical cultures. Cells from Sprague Dawley rats were cultured for 14 days before treatment with C9 for 4 consecutive days. The cells were analysed for protein expression of drebrin, MAP2, GFAP, glucose transporter type 4 (GLUT4), vesicular glutamate transporter 1 (vGluT1), and synapsin I using immunocytochemistry. The gene expression of related proteins was determined using qPCR, and viability assays were performed to evaluate toxicity. The results showed that protein expression of drebrin and MAP2 was increased, and the corresponding mRNA levels were decreased after treatment with C9 in the hippocampal cultures. The ratio of MAP2-positive neurons and GFAP-positive astrocytes was altered and there were no toxic effects observed. In conclusion, the IRAP inhibitor compound C9 enhances the expression of the pro-cognitive markers drebrin and MAP2, which further confirms IRAP as a relevant pharmaceutical target and C9 as a promising candidate for further investigation.

## 1. Introduction

The insulin-regulated aminopeptidase (IRAP; oxytocinase; placental leucine aminopeptidase) is part of the M1 aminopeptidase family and is highly expressed in many tissues, including the neocortex and hippocampus of the brain [1,2,3,4]. In 1995, IRAP was discovered in fat and muscle cells by Keller et al. [5] and has since been associated with various physiological functions, although the exact role of this peptidase remains unknown. It is involved in the degradation of several endogenous substrates, such as oxytocin, vasopressin, cholecystokinin-8 and somatostatin [3,6,7,8,9], and in the immune system where IRAP is involved in the major histocompatibility complex (MHC) class I antigen presentation and T cell activation [10,11]. IRAP can be found in glucose transporter type 4 (GLUT4) vesicles and is thought to mediate GLUT4 trafficking to the plasma membrane, indicating a potential role in regulating cellular glucose uptake [12,13,14,15]. Although IRAP has been shown to be involved in many different physiological functions, the evaluation of IRAP as a pharmaceutical target has mainly been focused on its role in the brain, more specifically in cognition [16,17]. At the beginning of the century, IRAP was identified as the receptor for the hexapeptide Angiotensin IV (Ang IV) [7,18]. Ang IV and related analogues have demonstrated enhanced memory effects in several behaviour models, as first demonstrated by Brazko et al. [19,20,21,22,23,24,25,26], and bind to the active site of the protein, thereby causing inhibition of its enzymatic activity. This inhibition is suggested to increase levels of peptides such as vasopressin and oxytocin and also modulate glucose uptake, leading to improved cognitive functions [6,7,12,14,15,27,28]. Reported data have initiated the search for new ligands with the capacity to inhibit IRAP in pursuit of cognitive enhancers that are more suitable as pharmaceuticals compared to the endogenous Ang IV and related peptides [16,29,30,31]. A series of macrocyclic compounds were synthesized and confirmed as IRAP inhibitors, and among those, compound 9 (C9) [32], exhibiting a similar inhibitory capacity as the structurally related HA08 [33], attracted our interest. Compounds C9 and HA08 combine structural elements from Ang IV and the physiological substrates oxytocin and vasopressin. A crystal structure of HA08 reveals that the macrocycle binds in the catalytic site in a near canonical substrate-like configuration and inhibits IRAP by a competitive mechanism [34]. Compound C9 encompasses a primary amide at the C-terminal rather than the acidic carboxylic acid function present in HA08. Despite the loss of the negative charge by replacement of the carboxyl group, the binding mode of C9 to IRAP seems unaltered as compared with HA08, according to a molecular dynamics analysis [32,33].

We have previously demonstrated that HA08 increases dendritic spine density and can reverse cellular viability in rat primary hippocampal cells [35,36]. Dendritic spines are small membrane protrusions on the neuronal dendrites and play an important role in synaptic connectivity by receiving and transmitting neuronal signals. Alterations in synaptic strength are linked to cognitive performance and different memory processes, and deviations in spine morphology or density are observed in neurodegenerative disorders, such as Alzheimer’s disease [37,38,39,40,41,42,43,44,45]. One important protein for defining and upholding the dendritic structure and synaptic transmission is the neuronal-specific marker microtubule-associated protein 2 (MAP2) [46]. MAP2 regulates cytoskeleton dynamics and intracellular trafficking and has been directly linked to the initiation of long-term potentiation by increasing its translocation to dendritic spines [47]. This microtubule protein is involved in many important physiological processes and is closely related to the neurodegeneration-related MAP Tau, although the pathological role of MAP2 remains to be determined [46]. In contrast to the neuronal marker MAP2, the astrocytic marker glial fibrillary acidic protein (GFAP) has been linked to several types of brain disorders and injuries [48]. Increased levels of GFAP in cerebral spinal fluid and plasma can be seen in patients with traumatic brain injuries and neuroinflammatory and neurodegenerative diseases. This astrocytic protein is therefore suggested as a potential biomarker and prognostic tool for some of the most common brain disorders, including Alzheimer’s and Parkinson’s disease [48,49].

To further evaluate potent IRAP inhibitors suitable for future pharmaceutical development, we have designed a study with a high-content screening approach to examine the impact of macrocycle inhibitor compound C9 on the dendritic spine-related protein drebrin and other key markers related to cognitive processes, such as neuronal marker MAP2, and astrocytic marker GFAP, in rat primary hippocampal and cortical cell cultures.

## 2. Results

### 2.1. The IRAP Receptor Is Expressed in Hippocampal and Cortical Cell Cultures

**Figure 1:**
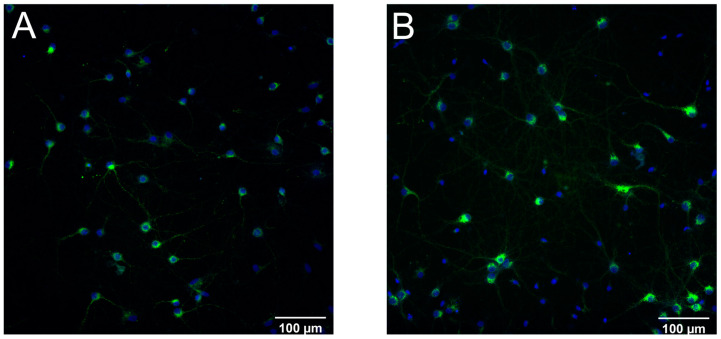
The expression of insulin-regulated aminopeptidase (IRAP) in primary hippocampal and cortical cell cultures. Untreated hippocampal and cortical primary cells were fixed at in vitro day 18 and immunocytochemistry was used to visualise the expression of IRAP, along with the cell nuclei (DAPI). The images were acquired using ImageXpress (Molecular Devices, San Jose, CA, USA ) with a 20× objective. (**A**) IRAP expression (green) and cell nuclei (blue) in untreated hippocampal cell cultures. (**B**) IRAP expression (green) and cell nuclei (blue) in cortical cell cultures.

Untreated primary cell cultures were analysed for the expression of IRAP at in vitro day 18 using immunocytochemistry (ICC). Both the hippocampal and cortical cultures were confirmed to express IRAP, see Figure 1A,B for representative images.

### 2.2. IRAP Inhibitor C9 Increases Drebrin Intensity

**Figure 2:**
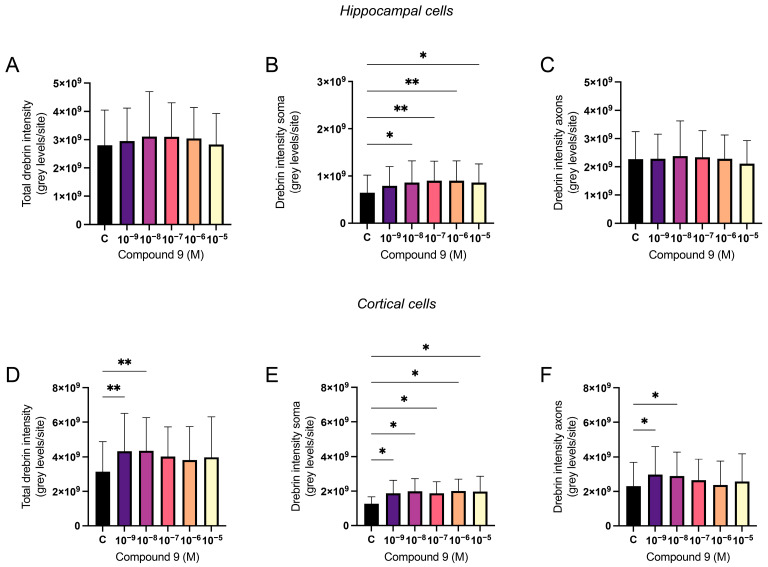
The effect of compound 9 (C9) on drebrin intensity in primary hippocampal and cortical cell cultures. The results of the dendritic spine density analysis for hippocampal and cortical cell cultures after four days of consecutive treatment with IRAP inhibitor C9. The vehicle group (C) was treated with 0.1% (*v*/*v*) DMSO. (**A**) The total drebrin intensity in hippocampal cells showed no significant overall treatment effect (*n* = 8). (**B**) The drebrin intensity in soma regions was significantly increased after treatment with 10^−^^8^, 10^−^^7^, 10^−^^6^, and 10^−^^5^ M C9 in hippocampal cells (*n* = 8). (**C**) The drebrin intensity in axons showed no significant overall treatment effect in hippocampal cells (*n* = 8). (**D**) The total drebrin intensity was significantly increased after treatment with 10^−^^9^ and 10^−^^8^ M C9 in cortical cells (*n* = 4). (**E**) The drebrin intensity in soma regions was significantly increased after treatment with 10^−^^9^, 10^−^^8^, 10^−^^7^, 10^−^^6^, and 10^−^^5^ M C9 in cortical cells (*n* = 4). (**F**) The drebrin intensity in axons was significantly increased after treatment with 10^−^^9^ and 10^−^^8^ M C9 in cortical cells (*n* = 4). All data are presented as means ± SD, * *p*-value < 0.05, ** *p*-value < 0.01.

**Figure 3:**
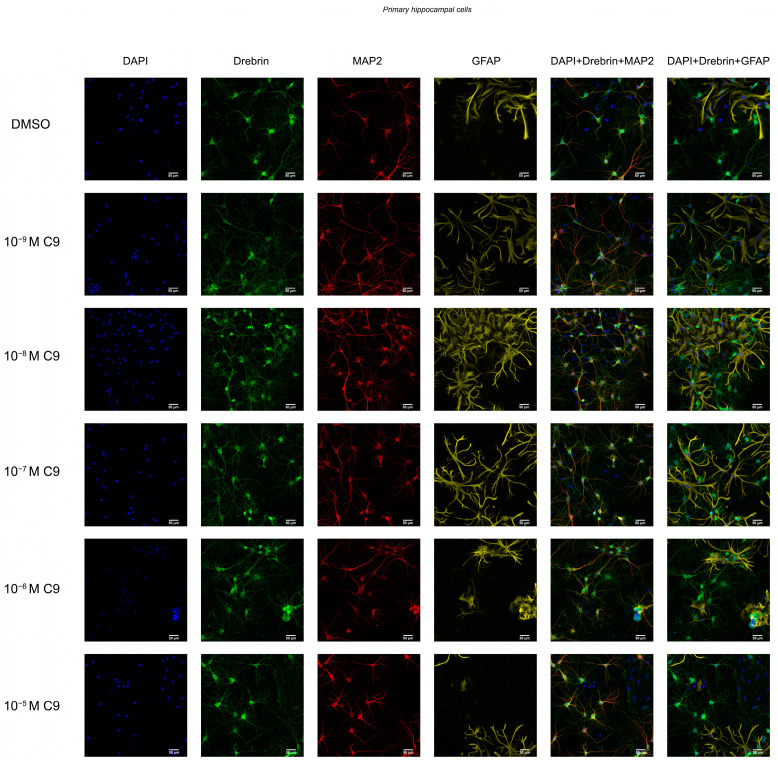
The drebrin expression in rat primary hippocampal neuronal cultures. Primary hippocampal cells were fixed at in vitro day 18 and immunocytochemistry was used to visualise drebrin along with the neuronal marker microtubule-associated protein 2 (MAP2), astrocytic marker glial fibrillary acidic protein (GFAP) and cell nuclei (DAPI). The fluorescent images represent the expression of drebrin (green), MAP2 (red), GFAP (yellow) and cell nuclei (blue) in hippocampal cell cultures after four days of consecutive treatment with IRAP inhibitor compound 9 (C9; 10^−^^9^, 10^−^^8^, 10^−^^7^, 10^−^^6^, and 10^−^^5^ M). The vehicle group was treated with 0.1% (*v*/*v*) DMSO, images shown in the top row. The images were acquired using ImageXpress (Molecular Devices) with a 20× objective.

**Figure 4:**
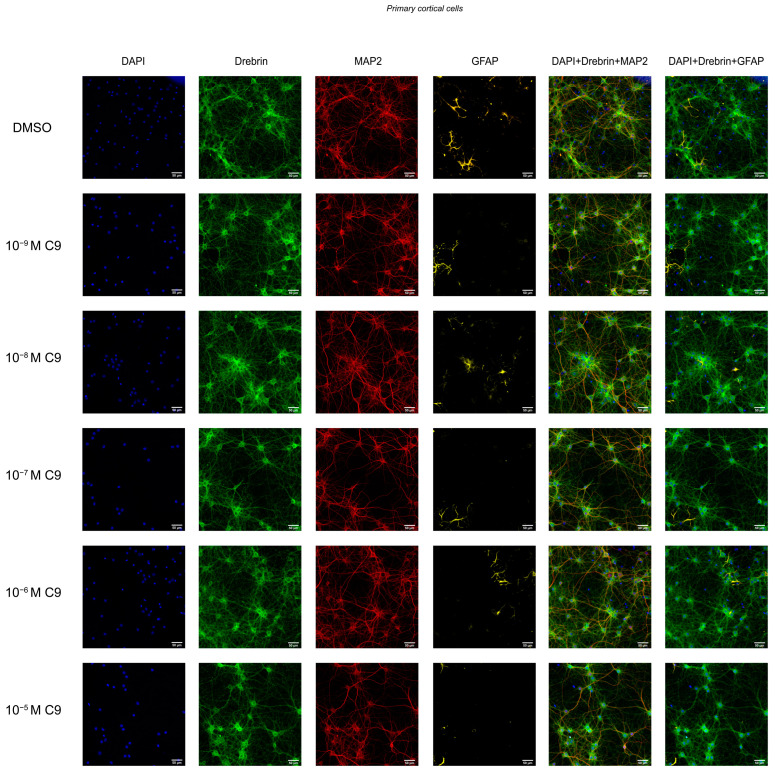
The drebrin expression in rat primary cortical neuronal cultures. Primary cortical cells were fixed at in vitro day 18 and immunocytochemistry was used to visualise drebrin along with the neuronal marker microtubule-associated protein 2 (MAP2), astrocytic marker glial fibrillary acidic protein (GFAP) and cell nuclei (DAPI). The fluorescent images represent the expression of drebrin (green), MAP2 (red), GFAP (yellow) and cell nuclei (blue) in cortical cell cultures after four days of consecutive treatment with IRAP inhibitor compound 9 (C9; 10^−^^9^, 10^−^^8^, 10^−^^7^, 10^−^^6^, and 10^−^^5^ M). The vehicle group was treated with 0.1% (*v*/*v*) DMSO, images shown in the top row. The images were acquired using ImageXpress (Molecular Devices) with a 20× objective.

The effect of repeated treatment with 1 nM to 10 µM C9 on the dendritic spine density was determined by measuring the fluorescence intensity of expressed drebrin in primary hippocampal and cortical cell cultures. The acquired images were analysed for total drebrin intensity, drebrin intensity in the soma region, and drebrin intensity in the axons. The average number of total cells analysed for each culture (*n* = 1) was approximately 3 × 10^4^ for the hippocampal cultures and 4.9 × 10^4^ for the cortical cultures. There was no overall effect of treatment on the total amount of drebrin intensity or the axonal drebrin intensity in the hippocampal cell cultures (Figure 2A,C). However, the soma drebrin intensity was significantly altered by the treatment (ANOVA *p*-value 0.0052, F-value 4.059) and post-hoc analysis revealed that 10^−^^8^, 10^−^^7^, 10^−^^6^, and 10^−^^5^ M C9 increased the amount of drebrin in the soma ROI of hippocampal cells when compared to the vehicle (0.1% (*v*/*v*) DMSO) treated group (*p*-values 0.0136, 0.0029, 0.0030, and 0.0141, respectively), see Figure 2B. The results of the C9 treatment in cortical cell cultures showed an overall treatment effect for total drebrin intensity (ANOVA *p*-value 0.0161, F-value 4.030) and soma drebrin intensity (ANOVA *p*-value 0.0192, F-value 3.846), as well as the axonal drebrin intensity (ANOVA *p*-value 0.0132, F-value 4.252), see Figure 2D–F. Post-hoc analysis revealed that the total amount of drebrin in the cells was significantly increased at concentrations 10^−^^9^ and 10^−^^8^ M C9 when compared to the vehicle treated cells (*p*-values 0.0073 and 0.0062), see Figure 2D. The soma drebrin amount was significantly increased for all concentrations of C9; 10^−^^9^, 10^−^^8^, 10^−^^7^, 10^−^^6^, and 10^−^^5^ M, when compared to vehicle (*p*-values 0.0352, 0.0120, 0.0365, 0.100, and 0.0141, respectively), see Figure 2E. The amount of drebrin located in the axons of the cortical cells was significantly increased at concentrations 10^−^^9^ and 10^−^^8^ M C9 when compared to vehicle (*p*-values 0.0103 and 0.0259), as shown in Figure 2F. See Figure 3 for representative images of drebrin expression in hippocampal cell cultures, and Figure 4 for representative images of cortical cell cultures.

### 2.3. Treatment with C9 Alters the Ratio of Neurons and Astrocytes

**Figure 5:**
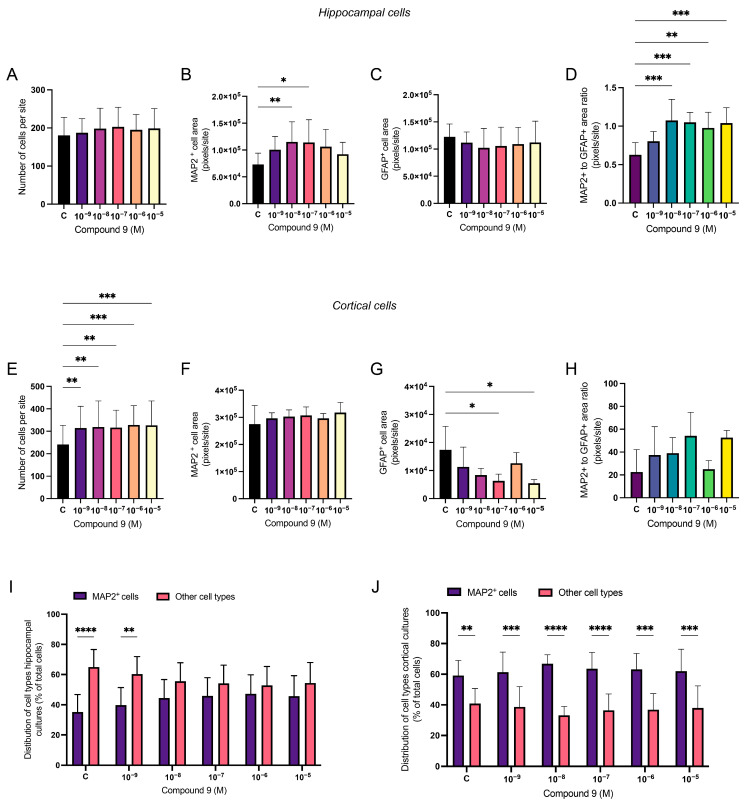
The effect of compound 9 (C9) on the distribution of cell types in primary hippocampal and cortical cells. The results of the cell type analysis for hippocampal and cortical cell cultures after four days of consecutive treatment with IRAP inhibitor C9. The vehicle group (C) was treated with 0.1% (*v*/*v*) DMSO. (**A**) There was no significant overall treatment effect of C9 on the total number of cells per site in hippocampal cultures (*n* = 8). (**B**) There was a significant increase in MAP2 cell area after treatment with 10^−^^7^ and 10^−^^5^ M C9, when compared to vehicle treatment, in hippocampal cultures (*n* = 6). (**C**) There was no significant overall treatment effect of C9 on the GFAP-positive cell area in hippocampal cultures (*n* = 4). (**D**) The pixel area ratio of MAP2 to GFAP-positive cells in hippocampal cultures was significantly increased after treatment with 10^−^^8^, 10^−^^7^, 10^−^^6^ and 10^−^^5^ M C9 (*n* = 4). (**E**) The total number of cells per site was significantly increased by all concentrations of C9, when compared to vehicle treatment, in cortical cells (*n* = 5). (**F**) There was no significant overall treatment effect of C9 on the MAP2-positive cell area in cortical cells (*n* = 4). (**G**) The amount of GFAP cell area was significantly increased after treatment with 10^−^^7^ and 10^−^^5^ M C9 compared to the vehicle group in cortical cells (*n* = 4). (**H**) There was no overall treatment effect of C9 on the pixel area ratio of MAP2 to GFAP-positive cells in cortical cultures (*n* = 4). (**I**) There was a significantly lower percentage of neurons in the vehicle-treated group, and the group treated with 10^−^^9^ M C9 in hippocampal cultures, compared to other cell types (*n* = 8). (**J**) There was a significantly higher percentage of neurons in all the treatment groups, compared to other cell types in cortical cultures (*n* = 6). All data are presented as means ± SD, * *p*-value < 0.05, ** *p*-value < 0.01, *** *p*-value < 0.001, **** *p*-value < 0.0001.

The ratio of neurons and astrocytes following treatment with 1 nM to 10 µM C9 in primary hippocampal and cortical cell cultures was determined using ICC. The acquired images were analysed for the total number of DAPI nuclei (total number of cells), the total pixel area of MAP2-positive cells (neurons), and the total pixel area of GFAP-positive cells (astrocytes). In addition to the pixel area, the number of MAP2-positive cells was also measured to determine the percental proportion of neurons in the cultures. The average number of cells analysed for each culture (*n* = 1) was approximately 2.8 × 10^4^ for the hippocampal cultures and 4.4 × 10^4^ for the cortical cultures. In the hippocampal cell cultures, there was no overall effect of C9 on the total number of cells per site (Figure 5A); however, there was an overall treatment effect on the MAP2 cell area per site (ANOVA *p*-value 0.0262, F-value 3.120), see Figure 5B. Further post-hoc analysis revealed that concentrations 10^−^^8^ and 10^−^^7^ M of C9 significantly increased the total area of cells positive for MAP2 when compared to the vehicle (0.1% (*v*/*v*) DMSO) treatment (*p*-values 0.0098 and 0.0120, respectively). The total pixel area of cells positive for GFAP was not significantly altered by treatment with C9 in the hippocampal cultures (Figure 5C). The area ratio of MAP2-positive cells to GFAP-positive cells was significantly altered by C9 treatment (ANOVA *p*-value 0.0002, F-value 10.55) and further post-hoc analysis revealed that 10^−^^8^, 10^−^^7^, 10^−^^6^, and 10^−^^5^ M of C9 increased the MAP2 to GFAP ratio (*p*-values 0.0002, 0.0003, 0.0017, and 0.0004, respectively), see Figure 5D. In the cortical cultures, there was an overall effect of C9 treatment on the total number of cells per site (ANOVA *p*-value 0.0005, F-value 7.175), along with the pixel area of GFAP-positive cells (ANOVA *p*-value 0.0238, F-value 3.626), see Figure 5E,G. Further post-hoc analysis revealed that all concentrations of C9, 10^−^^9^, 10^−^^8^, 10^−^^7^, 10^−^^6^, and 10^−^^5^ M, significantly increased the number of cells per site when compared to the vehicle (*p*-values 0.0019, 0.0011, 0.0014, 0.0003, and 0.0004). The area of cells positive for GFAP was significantly decreased by C9 treatment at concentrations of 10^−^^7^ and 10^−^^5^ M (*p*-value 0.0181 and 0.0109). There was no overall treatment effect of C9 on the pixel area of MAP2-positive cells nor on the area ratio of MAP2 to GFAP-positive cells (Figure 5F,H). The number of MAP2-positive cells (neurons) was calculated as a percentage of the total number of cells in both hippocampal and cortical cultures and compared to the percentage of other cell types (most likely different types of glial cells, like astrocytes). There was a significant difference between the percentage of neurons and percentage of other cell types in both hippocampal (ANOVA *p*-value < 0.0001, F-value 31.35) and cortical cultures (ANOVA *p*-value < 0.0001, F-value 94.40), and further post-hoc analysis revealed that there was a lower percentage of neurons in the vehicle-treated group and the group treated with 10^−^^9^ M C9 in hippocampal cultures, compared to other cell types (C; *p*-value < 0.0001, 10^−^^9^ M; *p*-value 0.0012), see Figure 5I. In the cortical cultures, all treatment groups showed a significantly higher percentage of neurons compared to other cell types (C; *p*-value 0.0058, 10^−^^9^ M; *p*-value 0.0008, 10^−^^8^ M; *p*-value < 0.0001, 10^−^^7^ M; *p*-value < 0.0001, 10^−^^6^ M; *p*-value 0.0001, and 10^−^^5^ M; *p*-value 0.0004), see Figure 5J.

### 2.4. GLUT4 Expression Is Increased in Cortical Astrocytes

**Figure 6:**
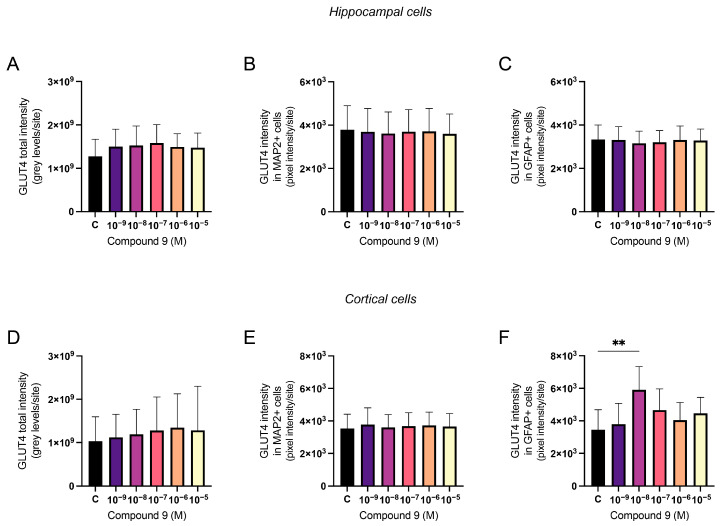
The effect of compound 9 (C9) on the expression of GLUT4 in primary hippocampal and cortical cells. The results of the GLUT4 analysis for hippocampal and cortical cell cultures after four days of consecutive treatment with IRAP inhibitor C9. The vehicle group (C) was treated with 0.1% (*v*/*v*) DMSO. There was no significant overall treatment effect of C9 on (**A**) the total GLUT4 intensity (*n* = 5); (**B**) the neuronal expression of GLUT4 (*n* = 5); or (**C**) the astrocytic expression of GLUT4 (*n* = 3) in hippocampal cells. There was also no significant overall treatment effect of C9 on (**D**) the total GLUT4 intensity (*n* = 5) and (**E**) the neuronal expression of GLUT4 (*n* = 5) in cortical cell cultures. (**F**) There was a significant increase in astrocytic expression of GLUT4 after treatment with 10^−8^ M C9, when compared to vehicle treatment, in the cortical cultures (*n* = 5). All data are presented as means ± SD, ** *p*-value < 0.01.

The effect of repeated treatment with 1 nM to 10 µM C9 on GLUT4 was determined by measuring the fluorescence intensity of expressed GLUT4 in primary hippocampal and cortical cell cultures. The acquired images were analysed for total GLUT4 intensity, GLUT4 intensity in MAP2-positive cells (neuronal GLUT4), and GLUT4 intensity in GFAP-positive cells (astrocytic GLUT4). The average number of cells analysed for each culture (*n* = 1) was approximately 3.7 × 10^4^ for the hippocampal cultures and 5.3 × 10^4^ for the cortical cultures. There was no overall effect of treatment on the total amount of GLUT4 intensity, the neuronal GLUT4 intensity divided with the total pixel area of MAP2-positive cells, nor on the astrocytic GLUT4 intensity divided with the total pixel area of GFAP-positive cells in hippocampal cultures (Figure 6A–C). In cortical cell cultures, there was no overall effect of treatment on the total GLUT4 intensity or the neuronal GLUT4 intensity per pixel area of MAP2-positive cells (Figure 6D,E). However, there was an overall treatment effect of C9 for the astrocytic GLUT4 intensity when divided with the pixel area of GFAP-positive cells (ANOVA *p*-value 0.0341, F-value 3.064). Further post-hoc analysis revealed that 10^−8^ M C9 increased the amount of GLUT4 intensity per GFAP-positive pixel (astrocyte area) in cortical cell cultures when compared to vehicle (0.1% (*v*/*v*) DMSO) treatment (*p*-value 0.0092), see Figure 6F.

### 2.5. Synaptic Markers vGluT1 and Synapsin I Are Not Significantly Altered by C9 Treatment

**Figure 7:**
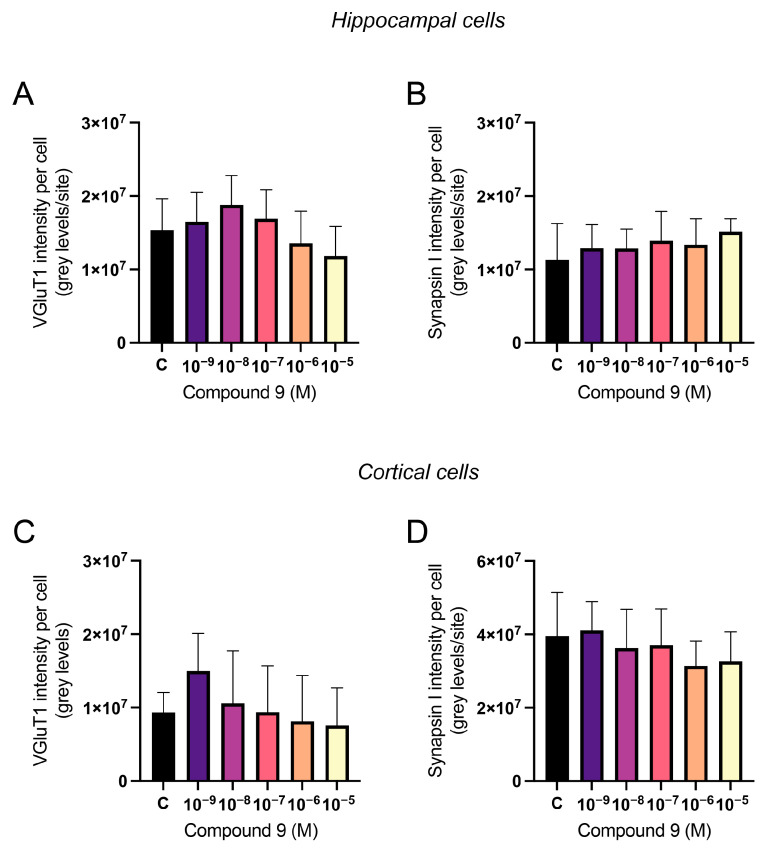
The effect of compound 9 (C9) on the expression of vGluT1 and synapsin I in primary hippocampal and cortical cells. The results of the vGluT1 and synapsin I analysis for hippocampal and cortical cell cultures after four days of consecutive treatment with IRAP inhibitor C9. The vehicle group (C) was treated with 0.1% (*v*/*v*) DMSO. (**A**) There was no overall treatment effect of C9 on the vGluT1 intensity per cell in hippocampal cultures (*n* = 5). (**B**) There was no significant overall treatment effect of C9 on the synapsin I intensity per cell in hippocampal cultures (*n* = 4). (**C**) There was a significant overall treatment effect of C9 on the vGluT1 intensity per cell in cortical cultures; however, further post-hoc analysis revealed no significant difference for any of the C9 concentrations when compared to vehicle-treated cells (*n* = 4). (**D**) There was a significant overall treatment effect of C9 on the synapsin I intensity per cell in cortical cultures, however further post-hoc analysis revealed no significant difference for any of the C9 concentrations when compared to vehicle-treated cells (*n* = 4). All data are presented as means ± SD.

The vesicular glutamate transporter 1 (vGluT1) and synapsin I expression were analysed in primary hippocampal and cortical cell cultures after treatment with 1 nM to 10 µM C9. The acquired images after ICC were analysed for the total number of cells (DAPI positive nuclei) and total amount of vGluT1 or synapsin I fluorescence intensity. The total intensity was divided by the total number of cells to normalise for differences in cell amount. The average number of cells analysed for each culture (*n* = 1) was approximately 2.8 × 10^4^ for the hippocampal cultures and 3.3 × 10^4^ for the cortical cultures. There was no overall treatment effect on the vGluT1 intensity per cell or the synapsin I intensity per cell in hippocampal cultures (Figure 7A,B). However, the treatment of C9 had an overall effect on the vGluT1 and synapsin I intensity per cell in cortical cultures (ANOVA *p*-value 0.0186 and F-value 3.980; ANOVA *p*-value 0.0327 and F-value 3.383, respectively). However, further post-hoc analysis revealed no significant difference in intensity for any of the C9 concentrations when compared to the vehicle (0.1% (*v*/*v*) DMSO) treated cells (Figure 7C,D).

### 2.6. IRAP Inhibitor C9 Decreases the mRNA Levels in Hippocampal Cells

The effects of repeated treatment with 1 nM to 10 µM compound C9 on the gene expression of *Dbn1*, *Gfap*, *Glut1*, *Glut3*, *Glut4*, *Lnpep*, and *Map2* were determined in primary hippocampal and cortical cell cultures by using qPCR. There was an overall treatment effect of C9 on the mRNA levels of *Dbn1* (ANOVA *p*-value 0.0083, F-value 6.289), *Gfap* (ANOVA *p*-value 0.0011, F-value 10.47), *Glut1* (ANOVA *p*-value < 0.0001, F-value 33.93), *Glut3* (ANOVA *p*-value 0.0319, F-value 4.116), *Glut4* (ANOVA *p*-value 0.0168, F-value 5.088), and *Map2* (ANOVA *p*-value 0.0379, F-value 3.872) in hippocampal cells (Table 1). Further post-hoc testing revealed that 10^−8^ and 10^−6^ M C9 decreased the gene expression of *Dbn1* when compared to vehicle (0.1% (*v*/*v*) DMSO) treated cells (*p*-value 0.0440 and 0.0035). There was also a significant decrease in *Gfap*, *Glut1*, and *Glut4* mRNA levels after treatment with 10^−8^, 10^−7^, and 10^−6^ M C9 (*Gfap p*-values 0.0085, 0.0126, and 0.0005; *Glut1 p*-values < 0.0001, 0.0008, and <0.0001; *Glut4 p*-values 0.0364, 0.0179, and 0.0296). The *Glut3* and *Map2* mRNA levels were significantly decreased at concentration 10^−6^ M C9 (*Glut3 p*-value 0.0170; *Map2 p*-value 0.0278). There was no overall treatment effect on the mRNA levels of *Lnpep*, the gene coding for IRAP, in hippocampal cells. However, in the cortical cell cultures, there was no overall treatment effect of C9 on any of the genes. See Table 1 for results of standardised mRNA levels of *Dbn1*, *Gfap*, *Glut1*, *Glut3*, *Glut4*, *Lnpep*, and *Map2* in hippocampal and cortical cell cultures.

### 2.7. Treatment with C9 Does Not Impact Viability of Primary Cell Cultures

**Figure 8:**
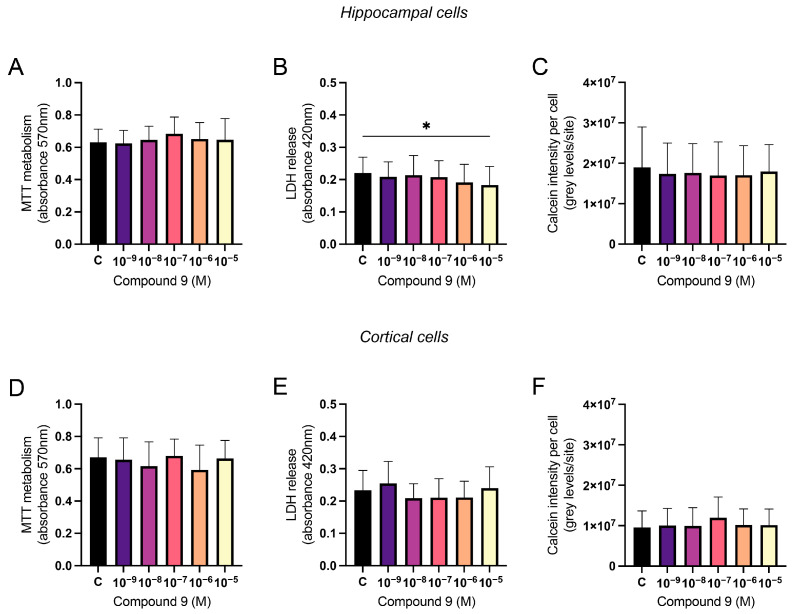
The effect of compound 9 (C9) on the cellular viability in primary hippocampal and cortical cells. The results of the MTT, LDH, and calcein metabolism analysis for hippocampal and cortical cell cultures after four days of consecutive treatment with IRAP inhibitor C9. The vehicle group (C) was treated with 0.1% (*v*/*v*) DMSO. (**A**) There was no significant overall treatment effect of C9 on the MTT metabolism in hippocampal cells (*n* = 7). (**B**) There was a significant decrease in LDH released in the cell media after treatment with 10^−5^ M C9, when compared to vehicle treatment, in hippocampal cells (*n* = 7). (**C**) There was no significant overall treatment effect of C9 on the calcein intensity per cell in hippocampal cultures (*n* = 4). (**D**) There was no significant overall treatment effect of C9 on the MTT metabolism in cortical cells (*n* = 7). (**E**) There was no significant overall treatment effect of C9 on the LDH release in cortical cells (*n* = 6). (**F**) There was no significant overall treatment effect of C9 on the calcein intensity per cell in cortical cultures (*n* = 4). All data are presented as means ± SD, * *p*-value < 0.05.

The mitochondrial metabolism, the membrane integrity, and the calcein metabolism were measured to assess the viability of the cell cultures after treatment with 1 nM to 10 µM C9. For the calcein assay, the average number of cells analysed for each culture (*n* = 1) was determined, and approximately 1.9 × 10^4^ cells were included in the data for each hippocampal culture and 2.7 × 10^4^ for each cortical culture. There was no overall treatment effect of IRAP inhibitor C9 in primary hippocampal or cortical cell cultures on the mitochondrial metabolism (Figure 8A,D). To determine the membrane integrity, the amount of LDH in the cell media was measured and for hippocampal cultures, there was an overall treatment effect of C9 (ANOVA *p*-value 0.0475, F-value 2.569). Further post-hoc analysis revealed that cells treated with the highest concentration of C9, 10^−5^ M, had a significantly lower amount of LDH leakage compared to vehicle (0.1% (*v*/*v*) DMSO) treatment (*p*-value 0.022), see Figure 8B. For the cortical cell cultures, there was no overall treatment effect on the release of LDH in the cell media (Figure 8E). The level of calcein metabolism in the cells was analysed by dividing the total calcein intensity by the number of cells per site (DAPI-positive nuclei). There was no overall treatment effect of C9 in the hippocampal cultures (Figure 8C) nor in the cortical cultures (Figure 8F).

## 3. Discussion

This study was designed for a high-content screening approach, where approximately 30–40 thousand cells were analysed for each culture, to study key markers related to cognitive processes in the brain. The results demonstrate that compound C9 increases the protein expression of drebrin, as well as alters the distribution of neurons (MAP2-positive cells) and astrocytes (GFAP-positive cells) in primary hippocampal and cortical cell cultures from rats. Interestingly, an unexpected result observed in this study was that there are prominent effect differences between hippocampal and cortical cultures.

MAP2 is a neuronal-specific marker that plays a key role in defining and upholding the dendritic structure. By influencing the microtubule cytoskeleton dynamics and intracellular trafficking, MAP2 regulates neuronal structure and synaptic transmission. It has also been directly linked to the induction of long-term potentiation, where the translocation of MAP2 to dendritic spines increased [47]. Dendritic spines are highly dynamic structures that are vital for neuronal plasticity and a key structure for cognitive functions [45]. There is also evidence suggesting that MAP2 is closely linked to the NMDA receptor; more specifically, NMDA receptor activation induces degradation of this microtubule protein [50]. Additionally, phosphorylation of MAP2 has been linked to the activation of insulin secretion upon glucose stimulation of CaM kinase II [51,52]. Both IRAP and MAP2 seem to be linked to the cellular processes of insulin signalling, as IRAP is located in GLUT4 vesicles in insulin-responsive cells, and is translocated and co-expressed with GLUT4 on the plasma membrane upon insulin stimulation [7,13]. Although MAP2 is involved in many important physiological processes, as well as closely related to MAP Tau, which is highly associated with neurodegenerative disorders, the pathological involvement of MAP2 is still unclear [46]. The findings from our study suggest that IRAP has an impact on the protein expression of MAP2 in primary hippocampal cultures from rats, indicating an involvement in the function and processes of dendritic spines and synaptic plasticity, which confirms previous studies were inhibition of IRAP increased dendritic spines [14,35,53]. Interestingly, the increase in MAP2-positive cells after treatment with compound C9 was only present in hippocampal cells. However, there was a significant decrease in GFAP-positive cells in the cortical cultures, which suggests that there is an impact on the neuron-to-astrocyte ratio in these cultures as well. GFAP is suggested as a potential biomarker for different brain disorders, including neuroinflammation and neurodegenerative disorders, like Alzheimer’s disease and Parkinson’s disease [48,49]. Decreased levels of this protein could therefore implicate an improved, or protective, effect on cellular health.

Glucose transporter deficiencies have been linked to disorders like Alzheimer’s disease, traumatic brain injury and stroke [54] and in IRAP knockout mice, the level of GLUT4 was markedly decreased [55]. Further, it is well known that IRAP is co-expressed with GLUT4 and that IRAP, therefore, is thought to regulate cellular glucose uptake by mediating GLUT4 trafficking to the cell membrane [12,13,14,15]. GLUT4 is believed to play a role in the mechanism behind memory improvement induced by IRAP inhibition [7,13,14], where enhanced formation of dendritic spines has been demonstrated to be dependent on GLUT4 [14]. The GLUT4 expression was therefore analyzed in our cell cultures following the C9 treatment, and the main effect on the amount of GLUT4 was linked to the GFAP-positive cells, the astrocytes, in the mixed primary cortical cultures. There was a significant increase in this glucose transporter in cortical astrocytes at 10 nM C9. IRAP is believed to be co-expressed with GLUT4 [2,13] and according to the results in this study, the amount of expressed GLUT4 remain approximately at the same level in untreated (vehicle treatment) hippocampal and cortical cells. However, the amount of expressed GLUT4 distributed in neurons and astrocytes remains the same in both hippocampal and cortical cultures (vehicle-treated cells), in contrast to the IRAP expression in the same type of untreated cells. A previous study on mouse hippocampus and cerebellum slices suggests that IRAP and GLUT4 are not always densely co-expressed and that this expression pattern might determine the function of IRAP and ligand inhibition [56]. They found that in the hippocampus, the co-expression was high and here Ang IV treatment induced an increase in glucose uptake, whereas in the cerebellum where the co-expression was minor, this effect on glucose uptake was not seen.

Surprisingly, the results of the mRNA analysis showed an opposing trend to what was seen from the protein expression analysis. The MAP2 protein expression was significantly increased in hippocampal cultures for all concentrations of C9, but the mRNA levels of the same protein, as well as for the gene encoding for drebrin, were significantly decreased at 1 µM. However, the results of the protein expression analysis are more essential to our conclusions, as the gene expression levels are not always fully reflected in cellular protein levels. The mRNA findings suggest a possible negative feedback, although future studies are needed to further explore this potential post-regulatory mechanism.

The results of the viability analyses, MTT, LDH, and calcein metabolism, did not demonstrate any toxic effects on hippocampal or cortical cell cultures after the administration of compound C9. On the contrary, there was a significant decrease in LDH release in hippocampal cells at 10 µM C9, suggesting that the membrane integrity was improved at this concentration. This suggests that some of the obtained data regarding an increased number of cells, or a change in distribution ratio between neurons and astrocytes after compound C9 treatment, could be a result of overall improved cellular health. This would be similar to previous findings, where we have seen that IRAP inhibition with HA08 can restore cell viability in rat primary hippocampal cultures after ROS-induced damage [36].

An unexpected result of this study was the findings demonstrating effect differences in the hippocampal and cortical cultures. The overall results demonstrate that more prominent effects were observed in the hippocampal cell cultures after IRAP inhibition. The analysis of the distribution of cell types in the cultures revealed that hippocampal cultures have a higher percentage of other cell types (GFAP-positive astrocytes and most likely other glial cells) compared to MAP2-positive neurons, whereas the cortical cultures revealed the opposite—a higher percentage of MAP2-positive neurons compared to other cell types. The differences in culture cell composition could explain the observed differences in MAP2 and GFAP protein expression, as these are cell-type-specific markers. We confirmed that IRAP is expressed in both culture types, although differences in the amount of expressed IRAP could also contribute to affect differences. A visual inspection of the fluorescent images suggests that cortical cultures have a slightly denser expression compared to the hippocampal cultures on in vitro day 18. Speculatively, the effects of IRAP inhibition could be reduced in cortical cultures as a consequence of a higher IRAP expression present to compensate.

We have chosen to use mixed primary cell cultures from rats to study the basic effects of the new IRAP inhibitor C9, as it is more similar to physiological conditions compared to a cell line. However, to further strengthen the conclusions on the IRAP inhibition effects on cognitive markers, additional functionality assays should be performed on these cultures, for example, to establish the presence of glutamatergic and GABAergic neurons. However, as the cells in our cultures are expressing vGluT1 and synapsin I, we draw the conclusion that there are active synapses and glutamate signalling present. Further, an additional limitation to the study is that the qPCR data are based on the entire cell culture composition, as opposed to the immunocytochemistry data where the actual protein expression of specific cell types (MAP2-positive neurons, GFAP-positive astrocytes) was measured. In addition to neurons and astrocytes, we know that there are also other types of cells in our cultures (most likely other types of glial cells), whose mRNA levels are also included in the qPCR analysis.

The aim of this study was to examine the effects of IRAP inhibitor C9 on key proteins linked to cognitive processes and to provide additional insight into the mechanisms behind the inhibition of IRAP and its cognitive enhancing effects. The present study demonstrates a strong relation between IRAP and the microtubule variant MAP2, and furthermore, the neurodegenerative disease marker GFAP is also altered by IRAP inhibition. Additionally, the impact of IRAP inhibition is proposed to be dependent on brain region and cell type. Further studies are needed to fully unravel the complex processes of cognitive functions and the role of IRAP in this. In conclusion, the IRAP-inhibiting compound C9 increases the expression of the pro-cognitive markers drebrin and MAP2, further confirming IRAP as a promising pharmaceutical target and compound C9 as a potent candidate for further investigation.

## 4. Materials and Methods

### 4.1. Animals

All animal experiments were performed in accordance with Swedish rules and guidelines for animal experiments (Animal Welfare Act SFS: 2018:1192) and the European Union directive on the Protection of Animals Used for Scientific Purposes (Directive 2010/63/EU). The animal experiment protocol was approved by the local animal ethics committee in Uppsala (permit number 5.8.18-18550/2018; 5.8.18-16657/2023). Foetuses of pregnant Sprague Dawley dams were used to set up primary cortical and hippocampal cell cultures.

### 4.2. Rat Primary Cell Cultures

The rat primary cell cultures were collected from embryonic day 17 Sprague Dawley foetuses by harvesting part of the cortex and the hippocampus, as described earlier [36]. Briefly, the tissue was digested and dissociated into a homogenous cell suspension and dissolved in Gibcos neurobasal plus media (NBM; Thermo Fisher Scientific, Waltham, MA, USA) supplemented with 0.25% glutaMAX™ (Thermo Fisher Scientific), 1% penicillin/streptomycin (Thermo Fisher Scientific) and 4% Gibcos B27 plus (Thermo Fisher Scientific). The cells were seeded on poly-D-lysine (Sigma–Aldrich, St. Louis, MO, USA) coated plates and kept in an incubator at 37 °C and 5% CO_2_.

### 4.3. IRAP Inhibitor Compound C9

The IRAP inhibitor used for treatment in the experiment was the synthetic macrocyclic substance compound C9 [32]. The IRAP inhibitor was dissolved in 100% dimethyl sulfoxide (DMSO) to a concentration of 10^−2^ M and stored at −20 °C. The stock solution was thawed and diluted into 10^−3^, 10^−4^, 10^−5^, 10^−6^, and 10^−7^ M solutions in 10% (*v*/*v*) DMSO for each experiment. During cell treatment, the inhibitor was further diluted 1:100 in cell media to final concentrations 10^−5^, 10^−6^, 10^−7^, 10^−8^, or 10^−9^ M in 0.1% (*v*/*v*) DMSO. The cells were grown for 13 days in vitro before C9 or vehicle (0.1% (*v*/*v*) DMSO) was added on days 14, 15, 16, and 17.

### 4.4. Immunocytochemistry

Immunocytochemistry (ICC) was used to visualize different cellular components after treatment with 10^−9^, 10^−8^, 10^−7^, 10^−6^, and 10^−5^ M C9. The dendritic spine-related protein drebrin was targeted to determine the effect on dendritic spines, along with the neuronal marker microtubule-associated protein 2 (MAP2) and astrocytic marker glial fibrillary acidic protein (GFAP). The glucose transporter 4 (GLUT4) was targeted in a separate ICC along with MAP2 and GFAP. ICC was also performed for synaptic markers vGluT1 and synapsin I. The cells were fixed with 4% PFA on in vitro day 18 and then permeabilized using 0.2% Triton-X 100. Normal donkey serum (10%) in 0.1% Triton-X 100 was added for 1h at room temperature (RT) to block unspecific binding before incubating the cells with primary antibody for the target of interest, also for 1h at RT. Primary antibodies rabbit anti-drebrin (Abcam, Cambridge, UK), mouse anti-MAP2 (Merck Millipore, Burlington, MA, USA), and rat anti-GFAP (Invitrogen, Waltham, MA, USA) were added at a concentration of 1:500. Primary antibodies rabbit anti-GLUT4 (Abcam), rabbit anti-vGluT1 (Abcam), and rabbit anti-synapsin I (Abcam) were added at a concentration of 1:250. The cells were then incubated for 1 h at RT with a secondary antibody (Alexa 488 anti-rabbit for drebrin, GLUT4, vGluT1, and synapsin I, Alexa 647 anti-mouse for MAP2, and Alexa 568 anti-rat for GFAP; Invitrogen) at a concentration of 1:500 and kept away from light from this point forward. This was followed by cell nuclei staining with 4′,6-diamidino-2-phenylindole (DAPI; Sigma–Aldrich) at a concentration of 2.5:500 for 25 min at RT. Additionally, untreated cells were fixed on in vitro day 18 to visualize the IRAP expression. For this ICC, the procedure was the same as described above, except Tween-20 was used as a permeabilization agent instead of Triton-X 100 to preserve the membrane-bound IRAP. Primary antibody rabbit anti-IRAP (D7C5; Cell signalling technology, Danvers, MS, USA) was added at a concentration of 1:250 and antibodies of mouse anti-MAP2 and rat anti-GFAP were added at a concentration of 1:500. Secondary antibodies used were Alexa 488 anti-rabbit for IRAP, Alexa 647 anti-mouse for MAP2, and Alexa 568 anti-rat for GFAP (Invitrogen).

### 4.5. Image Analysis

Fluorescently stained (ICC) 16-bit high-resolution images were acquired using a high-content screening device, ImageXpress (Molecular Devices,) mounted with a 10× objective. One cell plate represented one culture (*n* = 1), and for each treatment group, 27 different sites covering a minimum of 3 × 10^3^ cells were imaged from every culture (9 sites per well, three wells per treatment group). The images were analysed using an automated macro in ImageJ (version 1.52p), developed by the group. For all image analyses, the automated macros measured both intensity levels (grey levels) and area (pixels) of specific regions of interest (ROI). Intensity measurements are well suited for this type of semi-high content analysis. Images containing debris or other anomalies were removed from the analysis. Individual threshold values for each cell culture were set up at the beginning of the analysis using randomised images from the cell plate. Intensity was measured as integrated intensity (sum of all pixel intensities in a given ROI) if not stated otherwise. To ensure macro validity, the automated workflow was visually inspected during the first five minutes. For the dendritic spine analysis, images containing cell nuclei (DAPI), spine density (drebrin), and neuronal maturity (MAP2) were used. First, cells were detected and counted using the ImageJ built-in function “analyse particles”. Each cell was assigned a unique cell-ROI using the DAPI channel. The number of cell nuclei positive for MAP2 staining was reported, and cells with low MAP2-intensity were considered non-neuronal cells and were removed from further analysis. Second, the cell-ROI was dilated to capture the cell cytoplasm of the neurons and this new ROI (soma-ROI) was used to measure the intensity of drebrin around the cell nuclei and cell body, in which drebrin is more densely expressed. To avoid repeated measurement from cells located in close proximity to each other, the content within each soma-ROI was deleted after drebrin intensity measurement. Third, the drebrin intensity in the axons was defined by subtracting the value of the soma-ROI drebrin intensity from the total drebrin intensity in the analysed image. The remaining drebrin intensity value was defined as axonal drebrin. For the GLUT4 image analysis, the total intensity of GLUT4 was measured in both neurons (MAP2-positive cells), as well as astrocytes (GFAP-positive cells). First, a threshold was determined for channels MAP2 and GFAP, and the total area of respective channels was assigned an ROI. Second, an overlay of the MAP2-ROI was created on the GLUT4 channel and the intensity of GLUT4 in neurons was determined. Similarly, an overlay of the GFAP-ROI was created on the GLUT4 channel and the intensity of GLUT4 in astrocytes was determined. The area of these three channels was also reported. The data for the total cell area of the MAP2 and GFAP channels (collected from multiple ICC rounds) were also used to analyse the ratio of neurons and astrocytes in the cultures. For the calcein, vGluT1, and synapsin I analysis, images containing cell nuclei (DAPI) and either of the mentioned protein markers were used. These macros measured the number of cell nuclei and integrated the intensity of the protein signal in thresholded images.

### 4.6. mRNA Expression

Quantitative polymerase chain reaction (qPCR) was used to determine the gene expression (mRNA levels) after treatment with 10^−8^, 10^−7^, and 10^−6^ M C9. The primary cell cultures were lysed and homogenized on in vitro day 18 so that RNA could be extracted according to instructions of Qiagen RNeasy^®^ Plus Mini Kit (Cat.no 74134; Qiagen, Hilden, Germany). The RNA concentration was measured using a NanoDrop^®^ ND-1000 spectrophotometer (NanoDrop Technologies, Inc., Wilmington, DE, USA) and diluted to 20 ng/µL. The RNA samples were converted to cDNA using iScript cDNA synthesis kit (Bio-Rad Laboratories, Inc., Hercules, CA, USA). Every reaction sample contained 250 ng RNA, 5× iScript reaction mix, iScript reverse transcriptase, and RNase free water in a total volume of 20 µL. Negative control reactions were also included and contained no iScript reverse transcriptase. The samples were run according to the following protocol: 25 °C for 5 min, 46 °C for 20 min, and 95 °C for 1 min. The gene expression (mRNA levels) of *Dbn1*, *Gfap*, *Glut1*, *Glut3*, *Glut4*, *Lnpep*, and *Map2* was determined using quantitative polymerase chain reaction (qPCR) with SYBR^®^ Green (Bio-Rad Laboratories). *Actb*, *Rplp0*, and *Rpl19* were used as reference genes. Primers for each analysed gene were purchased from Thermo Fischer Scientific, see Table 2 for primer sequences.

The samples, including negative controls, were analysed in duplicates using a 96 well PCR plate. Each reaction sample contained 5 ng cDNA, 20 µM forward primer, 20 µM reverse primer, 1× iQ SYBR^®^ Green supermix (hippocampal cultures), or 1× SsoAdvanced Universal SYBR^®^ Green supermix (cortical cultures), and RNase free water in a total volume of 25 µL. The samples were run on the CFX96 real-time PCR detection system version 3.1 (Bio-Rad Laboratories) according to the following amplification protocol: 95 °C for 3 min and then 40 cycles of 95 °C for 15 s, 60 °C for 20 s, and 72 °C for 10 s. Each run ended with a melt curve to ensure specific amplification. The amplification efficiency of each primer set was obtained using linRegPCR software (version 2020.2) and the Cq-value of each sample was then obtained using qbase+ software version 3.4 (BioGazelle, Zwijnaarde, Belgium).

### 4.7. Mitochondrial Metabolism

To determine the mitochondrial activity in the cells after treatment with 10^−9^, 10^−8^, 10^−7^, 10^−6^, and 10^−5^ M C9, tetrazolium bromide salt (MTT; Sigma–Aldrich) was used. On in vitro day 18 the cells were incubated with MTT (0.8 mg/mL) for 30 min at 37 °C in the dark after which the cells were lysed with 100% DMSO and then incubated in the dark for 15 min at RT. Triton-X 100 was used as a negative control for mitochondrial metabolism. Active mitochondria metabolised the added MTT to a purple formazan product, and the amount of developed formazan product corresponds to the amount of metabolised MTT. The plate was measured in a plate reader (FLUOstar Omega, Ortenberg, Germany) for absorbance at 570 nm. All plates analysed for mitochondrial activity had a cell density of approximately 5 × 10^4^ cells per well.

### 4.8. Membrane Integrity

To determine the membrane integrity of the cells after treatment with 10^−9^, 10^−8^, 10^−7^, 10^−6^, and 10^−5^ M C9, the amount of lactate dehydrogenase (LDH) in the cell media was measured using a cytotoxicity detection kit (Sigma–Aldrich). When the cell membrane is damaged, the cytoplasmic LDH leaks out in the cell media. The LDH reagent mix was added to the cell media on in vitro day 18 and then incubated for 30 min in the dark at RT. Triton-X 100 was used as a positive control for cell toxicity. A red formazan product is produced when there is LDH present in the cell media and the amount of formazan product developed corresponds to the amount of LDH. The plate was measured in a plate reader (FLUOstar Omega) for absorbance at 492 nm. All plates analysed for membrane integrity had an approximate cell density of approximately 5 × 10^4^ cells per well.

### 4.9. Calcein Metabolism

The amount of metabolised calcein was measured in the cells as part of the viability assessment of treatment with 10^−9^, 10^−8^, 10^−7^, 10^−6^, and 10^−5^ M C9. In cells with high viability, a high metabolism is present where intracellular esterases are part of this activity. Calcein acetoxymethyl (AM; Invitrogen, Thermo Fischer Scientific) is a cell-permeable compound that is cleaved by active esterases, which produce a fluorescent dye. Calcein AM, 1 µM, was added to the treated cells together with fresh media on in vitro day 18 and incubated for 40 min at 37 °C in the dark. The calcein media was then removed and 5 µg/mL Hoescht (Invitrogen, Thermo Fischer Scientific) was added to the cells together with 1× PBS and incubated for 15 min at 37 °C in the dark before images were acquired of the fluorescent metabolised calcein and the cell nuclei. Images were acquired using ImageXpress (Molecular Devices). All plates analysed for calcein metabolism had a cell density of approximately 1.5 × 10^4^ cells per well.

### 4.10. Statistical Analysis

GraphPad Prism (version 10.2.3) was used to perform all statistical analysis. Tissues collected from foetuses of one individual Sprague Dawley dam were considered as one primary cell culture (*n* = 1). Raw data comparing multiple groups (>2) were analysed using two-way ANOVA with treatment and culture as factors. If there was a significant overall treatment effect, further post-hoc testing was performed using Dunnett’s post-hoc test with comparison to the vehicle-treated group (0.1% (*v*/*v*) DMSO). Data converted to percentages comparing multiple groups (>2) were analysed using one-way ANOVA. If there was a significant overall treatment effect, the one-way ANOVA was followed by a Dunnett’s post-hoc test with comparison to the vehicle-treated group. Data where two groups were compared were analysed using an unpaired *t*-test. All data is visualised as means ± standard deviation (SD) and statistical significance was defined as *p*-value < 0.05.

## Figures and Tables

**Table 1:** The mRNA levels of *Dbn1*, *Gfap*, *Glut1*, *Glut3*, *Glut4*, *Lnpep*, and *Map2* in rat primary hippocampal (*n* = 3–5) and cortical (*n* = 3) cell cultures presented as the mean of the percentage of control (vehicle-treated group). The cells were treated with compound 9 (C9) (0.01, 0.1, and 1 µM) for four consecutive days (vehicle treatment 0.1% DMSO) before being analysed for mRNA levels using qPCR.

Standardised mRNA Levels of *Dbn1*, *Gfap*, *Glut1*, *Glut3*, *Glut4*, *Lnpep* and *Map2* ^1^							
Gene:	*Dbn1*	*Gfap*	*Glut1*	*Glut3*	*Glut4*	*Lnpep*	*Map2*
C9 0.01 µM	61.1 * ±31.9	70.2 ** ±12.2	55.7 **** ±11.3	67.2 ±15.5	58.1 * ±14.4	86.3 ±27.9	71.0 ±13.0
C9 0.1 µM	70.5 ±24.6	74.3 * ±17.7	76.8 *** ±8.7	72.1 ±30.4	56.3 * ±22.3	104.7 ±30.2	92.5 ±32.6
C9 1 µM	46.2 ** ±13.8	60.8 *** ±8.7	62.4 **** ±5.4	57.6 * ±19.6	60.0 * ±30.8	69.3 ±13.4	63.4 * ±11.3
C9 0.01 µM	87.8 ±11.6	85.4 ±33.6	101.5 ±37.6	75.9 ±17.9	77.6 ±30.5	95.5 ±29.4	99.7 ±40.2
C9 0.1 µM	103.3 ±32.9	102.2 ±21.9	99.1 ±23.7	84.6 ±5.4	88.0 ±20.8	84.1 ±12.5	82.7 ±12.7
C9 1 µM	72.4 ±23.6	126.8 ^2^ ±6.0	101.5 ±22.4	88.2 ±15.3	73.8 ±18.9	87.7 ±11.7	94.4 ±17.5

Data presented from the hippocampal cell cultures are marked in light grey, and data presented from the cortical cell cultures are marked in grey. ^1^ All data are presented as means of percentage of control ± SD and were analysed using one-way ANOVA. The significant result of the ANOVA was followed by post-hoc analysis with Dunnett’s multiple comparison test comparing C9-treated cells to vehicle-treated cells. * *p*-value < 0.05, ** *p*-value < 0.01, *** *p*-value < 0.001, **** *p*-value < 0.0001. ^2^ Analysis of *Gfap* mRNA levels after treatment with C9 1 µM in cortical cell cultures *n* = 2.

**Table 2:** The mRNA levels of *Dbn1*, *Gfap*, *Glut1*, *Glut3*, *Glut4*, *Lnpep*, and *Map2* were analysed using qPCR in primary hippocampal and cortical cell cultures after treatment with IRAP inhibitor compound C9. *Actb*, *Rplp0*, and *Rpl19* were used as reference genes. Forward (FW) and reverse (R) primer sequences for each gene are presented in the table below.

Protein	Gene	Primer Sequence
Beta-actin	*Actb*	FW: CGTCCACCCGCGAGTACAACCT R: ATCCATGGCGAACTGGTGGCG
Ribosomal protein lateral stalk subunit P0	*Rplp0*	FW: GGGCAATCCCTGACGCACCG R: AGCTGCACATCGCTCAGGATTTCA
Drebrin	*Dbn1*	FW: TCAGACAGCAGGAACGAGTG R: TATGAAAGGGCAGTACGGACG
Glial fibrillary acidic protein	*Gfap*	FW: GTGGTATCGGTCCAAGTTTGC R: GACTCAAGGTCGCAGGTCAA
Glucose transporter type 1	*Glut1*	FW: GAGCTAGGAGGCTTTACCGC R: CCTAAATGGAGCCTGGACCC
Glucose transporter type 3	*Glut3*	FW: ATGGGGACAGCGAAGGTGAC R: CCCCTCGCTTGGTAGGTCTT
Glucose transporter type 4	*Glut4*	FW: AGGCCGGGACACTATACCC R: TAGCCAAACTGAAGGGAGCC
Insulin-regulated aminopeptidase	*Lnpep*	FW: GAGTGACAAAGACCGAGCCA R: TCTGAAGAGGCACTTTGCCA
Microtubule-associated protein 2	*Map2*	FW: GACCACCAGGTCAGAACCAAT R: TGGGCACCAAGATGCCAAAT
Large ribosomal subunit protein eL19	*Rpl19*	FW: GCGTCTGCAGCCATGAGTATGCTT R: ATCGAGCCCGGGAATGGACAGT

## Data Availability

The data presented in this article are not readily available because the data are part of an ongoing study and due to technical limitations. The raw data supporting the conclusions of this article can be made available by the authors on request.

## Abbreviations

Ang IV	Angiotensin IV
ANOVA	Analysis of variance
DAPI	4′,6-diamidino-2-phenylindole
DMSO	Dimethyl sulfoxide
GFAP	Glial fibrillary acidic protein
GLUT	Glucose transporter
ICC	Immunocytochemistry
IRAP	Insulin-regulated aminopeptidase
LDH	Lactate dehydrogenase
MAP2	Microtubule-associated protein 2
MTT	Tetrazolium bromide salt
ROI	Region of interest
SD	Standard deviation
vGluT1	Vesicular glutamate transporter 1
